# Growth, Spectroscopy, and Laser Performance of a 2.79 μm Er: YSGG Single Crystal Fibers

**DOI:** 10.3390/ma17020429

**Published:** 2024-01-15

**Authors:** Baiyi Wu, Meng Wang, Jian Zhang, Zhitai Jia, Zefeng Wang

**Affiliations:** 1 College of Advanced Interdisciplinary Studies, National University of Defense Technology, Changsha 410073, China; 18769783256@163.com (B.W.);; 2 Nanhu Laser Laboratory, National University of Defense Technology, Changsha 410073, China; 3 State Key Laboratory of Crystal Materials, Shandong University, Jinan 250100, China

**Keywords:** Er-doped crystal, single crystal fiber, 3 μm laser

## Abstract

Single crystal fibers combine the great specific surface area of fibers and the single crystal property of the bulk crystal which shows great potential for a high-power laser. For an Er-doped crystal, due to the fluorescence quenching at the 3 μm wavelength, high Er doping is necessary to increase the fluorescent up-conversion for the breaking limitation. However, a high Er doping concentration must lead to high heat accumulation, resulting in poor laser performance. Compared with an Er-doped bulk crystal, Er-doped SCF has the great potential to remove the heat in the crystal, and it is easy to obtain a high power. In this paper, Er: Y_3_Sc_2_Ga_3_O_12_ (Er: YSGG) single crystals were successfully grown using the micro-pulling-down method (μ-PD). Owing to the stably grown interface, the diameter of the crystal is 2 mm with a length up to 80 mm. Then, the measurements of Laue spots and Er^3+^ distribution indicated that our crystals have a high quality. Based on the as-prepared Er: YSGG SCF, the continuous-wave (CW) laser operations at 2794 nm were realized. The maximum output was 166 mW with a slope efficiency of up to 10.99%. These results show that Er: YSGG SCF is a suitable material for future high-power 3 μm laser operation.

## 1. Introduction

Solid-state lasers operating in the mid-infrared band show great prospects in laser surgery, environment detection, optical oscillation pump and the military field. Particularly, 2.7–3.0 µm lasers are important to optical parametric oscillation (OPO) for obtaining 3–15 µm lasers. Such lasers can be obtained with Er-doped crystals due to the operation on the Er^3+^: ^4^I_11/2_→^4^I_13/2_ transition [1,2,3,4]. However, there are still several issues in Er-doped crystals that limit its application. Firstly, the absorption efficiency of pumping light is usually too low due to the weak absorption peak of Er at 940 nm. As a result, some suitable sensitized ions like Yb^3+^ or Cr^3+^ need to be co-doped to enhance the absorption of the pump [5,6,7,8]. Secondly, the lifetime of the lower energy level is much longer than that of the upper level, which leads to self-terminating behavior and low laser efficiency. As we know, population inversion is requisite for laser generation, if the lifetime in the lower-level is greater than that in the high-level will lead the population of particles to gather in the lower level and the transition from the higher level to the lower level will be limited. Enough population inversion can be achieved with two methods. One is that a very high doping concentration will provide enough particles, and the other solution is accelerating the relaxation of particles in the lower level to make the transition to Er^3+^: ^4^I_11/2_→^4^I_13/2_ has a higher probability of maintaining the laser operation. For Er-doped crystals, decreasing the lifetime of ^4^I_13/2_ is an important way to promote laser output [9,10,11].

Presently, 50 at. % Er: YAG is a traditional gain medium to use due to its easy preparation and high and stable laser operation. However, the high doping concentration of the crystal will often make it hard for the crystal growth to obtain high-quality samples and have a serious thermal effect during operation. Like YAG, YSGG also has excellent thermal conductivity. Compared with a YAG crystal, its emission at 2.79 μm can be transmitted through a silica fiber with lower loss. The phonon energy of a YSGG crystal is also relatively lower, and it can help with a high doping concentration and a reduction in the probability of multi-phonon relaxation. Moreover, the segregation coefficient is close to 1, showing the high optical quality of Er: YSGG, and homogeneous distribution of Er^3+^ can be easily obtained using the melt method [12]. Therefore, much attention has been drawn to the laser operation of Er: YSGG. The maximum CW laser output in 2.79 μm is 1.8 W, and the slope efficiency is 11.2%. Nie et al. improved the slope efficiency by up to 31% with an Er: YSGG sample grown using the Czochralski method [13]. However, the laser power was limited by the thermal effect, so the result still needs to be optimized. Prior research has thoroughly investigated and proved that the key factors to achieve a high laser output are applicable crystal materials and the thermal effect management in high-power-output devices. Hence, it is important to improve the thermal management level of crystals through innovating fabrication technology.

A single crystal fiber (SCF) is a novel solid gain medium for further high laser output. Compared with traditional bulk crystals, a high aspect of the laser gain medium, for example, glass fiber, can be beneficial to the thermal management of a high laser output system. Meanwhile, because a low loss in the mid-infrared and high doping concentration can be achieved in SCF, the laser output of SCF will be higher than that of glass fiber, but the length of the used SCF can be much shorter. Therefore, it would be an applicable gain medium material to achieve the desired wavelength laser. More importantly, heavily doped and high-quality single crystal fibers can be prepared easily [7,13]. With the development of a growth method and SCF laser, the various crystals were prepared, and traditional garnet crystal-like YAG was the most studied material, with different doping rare earth elements applied for the laser system in the range of 1–2 μm. The modification of the material for a higher laser output and longer wavelength is still a hot topic in this field [14,15,16,17,18,19]. The common method for SCF growth is laser-heated pedestal growth (LHPG) and micro-pulling-down (μ-PD); the feature of LHPG is that the CO_2_ laser heating and high precision setup can help to grow a high-melting-point and extreme-shaped crystal because it is crucible-free. The design of the thermal field is the key to achieving the high-quality crystal. As we know, the laser focus is limited, so the large temperature gradient will lead to cracking. The most significant features of μ-PD are that the thermal field around the growing interface can be controlled by the design of the crucible, the after heater and the thermal insulations; the growth rate of SCFs can reach up to 4–5 mm/h with high efficiency. The crucible and after heater made with a high-melting-point material are the key factors for crystal growth, and more importantly, the length of SCFs can reach up to several centimeters with few raw materials. The performance of the laser based on bulk crystal and glass fiber has developed over several years; the laser structure of the bulk crystal laser and glass fiber laser are stable and mature, but the improvement in the laser output is limited by the gain medium. In recent years, some researchers have started to find a new gain medium to optimize the laser structure. SCF was selected as a new concept to solve the problem. Although the laser output of SCF is not as high as in the bulk crystal, the thermal management, dimension of crystal and cavity length in SCF also show potential applications in laser performance. In order to develop the SCF laser, there two different laser structures were given. One learned from the development of a traditional solid-state laser, which is characterized by a crystal fiber with a diameter of 0.4–1 mm, and the SCF is air-cladded. The waveguide is formed by multi-mode pumping of light into the SCF, and the two end cavity mirrors can ensure the oscillation of the signal light, which usually has a high output laser beam quality. At present, this method can achieve better laser output results. The other is to directly utilize the glass fiber laser; the crystal core was wrapped by a lower refractive index material to form a double-clad fiber to achieve a wavelength structure, and the cladding can be made from a silica fiber, multi-hole Al_2_O_3_, special glass and so on. The diameter of this SCF core is 20–100 μm, and the crystal is flexible and bendable [20,21]. The laser setup in this work was carried out using the first option.

In this work, 30 at% Er: YSGG SCF with a diameter of 2 mm was successfully grown by the μ-PD. Then, the result of crystallization property and concentration distribution of Er showed that the as-grown crystal has high-quality. Furthermore, the relevant investigation of the fluorescence emission spectra and fluorescent lifetime were calculated simultaneously. Lastly, the continuous-wave (CW) laser operations at 2794 nm were realized with the maximum output of 166 mW. Our works proved that the Er: YSGG SCFs are promising materials for the future 3 μm laser generation.

## 2. Materials and Methods

### 2.1. Single Crystal Fiber Growth

We prepared the raw materials using a conventional solid-state reaction. After the raw materials were mixed and sintered in air at 1350 °C for 48 h, we obtained the Er: YSGG polycrystalline materials. In order to keep stoichiometric proportion due to the loss of volatilization during growth, we should add an excess of 2 wt.% Ga_2_O_3_ when we prepare the raw materials [22,23]. The formula of the synthetic reaction is shown below:

3x Er_2_O_3_ + 3(1 − x) Y_2_O_3_ + 2 Sc_2_O_3_ + 3 Ga_2_O_3_ = 2 (Er_x_Y_1−x_)_3_Sc_2_Ga_3_O_12_ (0 < x < 1)

The YSGG SCFs were grown using the μ-PD method using a furnace with shock chamber to decrease mechanical vibration. In our experiment, a high-quality Er: YSGG crystal oriented to <111> was selected as seed crystal. A Φ 3 mm die Ir crucible was applied to contain and melt the polycrystalline materials. Moreover, Argon and CO_2_ with high-purity were used as the growth atmosphere to protect the Ir crucible. During the growth, we can clearly observe the as-grown fiber with a CCD camera and can adjust the parameter to control the liquid–solid interface in real-time. The raw materials were heated by RF, and when we noticed the molten YSGG suspending in the bottom of crucible, the raw materials were totally melted. The seed crystal which was fixed on a stage was controlled to touch the crucible to start crystal growth. The conditions of crystal growth were studied in this section. The temperature in the system is most important; when the seed crystal touched the bottom of crucible, the melt flowed by the pulling of seed crystal. The higher temperature will make the end crystal grow after few crystals have grown, because low viscosity will break the balance of the gravity and surface tension, so a necessary condition for high-quality crystal growth is that the growth temperature is close to or a little lower than the melting point. When the temperature and liquid–solid interface were stable, we started the growth with the pulling rate of 4–5 mm/h.

### 2.2. Crystal Quality of YSGG

X-ray Laue Back-Reflection system (Multiwire Laboratories, MWL120, Lansing, NY, USA) was performed to characterize the crystallinity by comparing the Laue spots in different positions, which can reflect the crystallinity and orientation of YSGG; the properties of crystallinity and orientation are vital measurements of crystal quality. Moreover, the Electron Probe microanalysis (EPMA, Shimadzu, 1720H, Kyoto, Japan) was used to measure the distribution of rare earth elements, and X-ray fluorescence analysis was employed to determine the actual ratio of the elements. The composition of doped crystal will change with raw materials because of the segregation effect and volatilization during growth at such high temperatures, so the distribution of rare earth elements, especially at high doping concentration, will influence the further laser experiment. The polished crystals with the dimensions of Φ 2 × 3 mm^3^ were used as the characterized samples. A Physical Property Measurement System (PPMS) was employed to measure the thermal conductivity of the YSGG sample in the range of 273–383 K. The thermal conductivity of crystal will reduce with the temperature increasing during the laser operation which makes the laser efficiency reduce and causes damage to crystal, so we measured the tendency of thermal conductivity in the range of 273–383 K to verify the reliability of crystal in laser operation. Moreover, the samples were used to measure the spectral performance under a 970 nm LD with a spectrophotometer, and emission and fluorescence decay curves were carried to optimize the laser setup.

### 2.3. Laser Experiment

**Figure 1:**
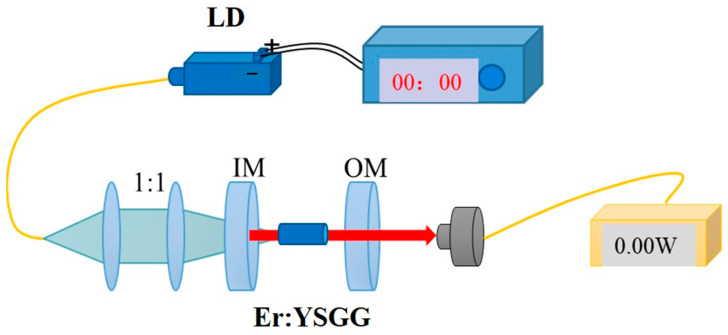
Laser experiment setup. Laser diode (LD); input mirror (IM); output mirror (OM).

In order to explore the laser performance of the as-prepared SCF, we designed a laser experiment to obtain the laser output around 3 μm. The experimental setup of continuous-wave (CW) laser experiment is shown in Figure 1. Before the experiment, we measured the absorption spectra of Er: YSGG. In order to obtain high efficiency laser output, we selected a 976 nm fiber-coupled laser diode as pump source. In this experiment, owing to the high-gain property of SCF, high doping concentration is not necessary, so 30 at% Er: YSGG was used to obtain the desired wavelength laser output.

## 3. Results and Discussion

**Figure 2:**
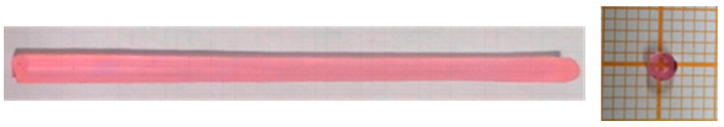
As-grown 30 at. % Er: YSGG.

Er: YSGG SCFs with diameter of 2 mm were successfully obtained using the μ-PD method. The SCFs length can reach up to 100 mm, and the color of the crystals was pink (Figure 2). Because of the volatilization of Ga_2_O_3_, the surface of the Er: YSGG showed non-transparent relatively, while the cross section was transparent without any volatiles. As the growth rate was changed from 1 mm/h to 5 mm/h, and mixed gas atmosphere mentioned previously was used to restrain volatilization of Ga_2_O_3_, the quality of the crystal was further optimized. The optimized fast growth speed is beneficial to decrease the corrosion of gallium since the as-grown crystal can deviate from the growing region rapidly. The XRF result (Table 1) showed that the components of crystal were (Er_0.27_Y_0.71_)_3_Sc_2_Ga_3_O_12_, which was close to the calculated stoichiometric ratio. The segregation coefficient of Er is 0.9 which is close to 1; this conclusion can provide a convenient and useful method to grow high-doping and volatile crystals.

**Figure 3:**
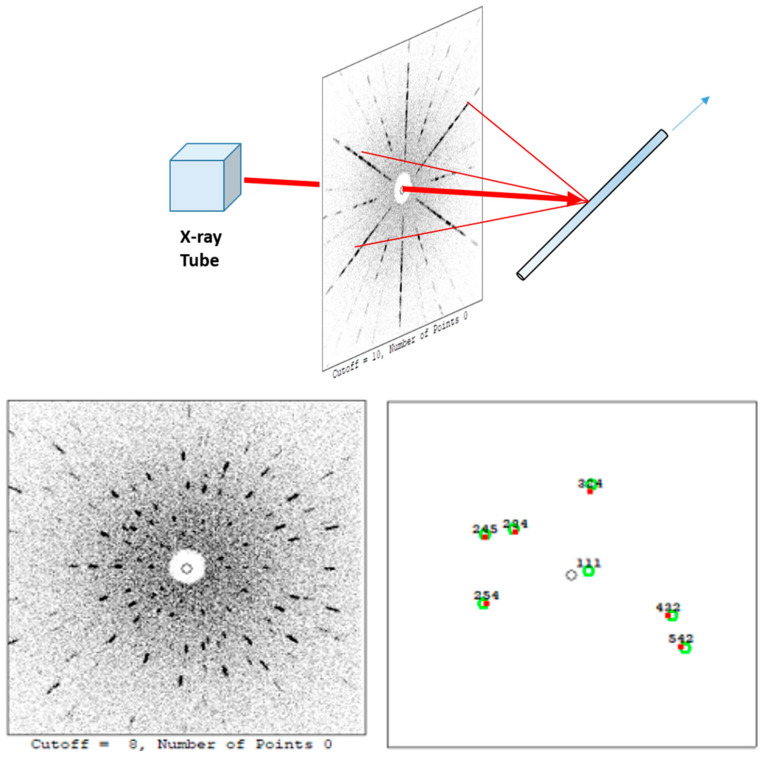
Laue patterns and the result of orientation.

The quality of YSGG was evaluated using the properties of crystallinity, thermal conductivity, optical homogeneity and concentration distribution along the axial direction. The results of the measurements are as follows. Er: YSGG with the dimensions of Φ 2 × 10 mm^3^ was well polished to measure the Laue spot along the crystal. We used an X-ray Laue Back-Reflection system to obtain Laue spots, and the end face of the polished crystal was fixed on the three-axis stage. Considering the dimension of SCF, the distance of the sample and tube is 125 mm. When the single crystal is exposed to continuous X-ray irradiation, it will generate a regular distribution pattern known as spots on the photographic plate. As seen in Figure 3, the Laue spots are legible and well aligned. It means that Er: YSGG SCFs have excellent crystallinity and a high quality.

**Figure 4:**
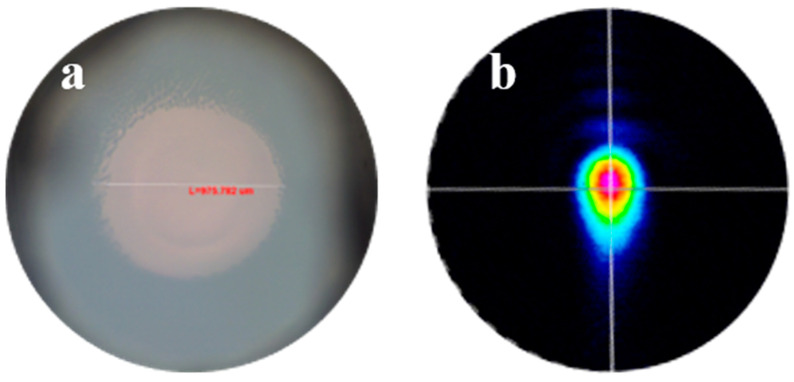
Optical quality of YSGG: (**a**) cross section with microscope; (**b**) beam spots across the crystal (the sample is <111> orientation crystal).

**Figure 5:**
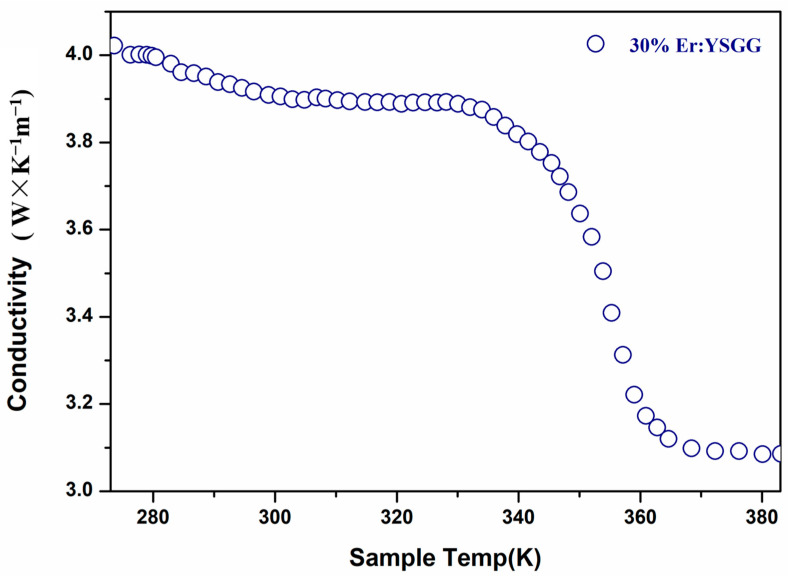
Thermal conductivity of the YSGG at 273–383 K.

Optical transmission properties of YSGG were measured with a laser beam system using a He-Ne laser source [14]. The laser beam regulated by the diaphragm was passed through the polished crystal samples, and then, the laser spots were monitored with a detector. The beam spot and analysis of the degree of distortion are shown in Figure 4. The result showed that the laser beam was driven into the crystal, but the quality still remains, showing that the optical property of the as-grown crystal is still of high quality. As we know, the thermal effect is one of main problems limiting the laser output, so it is of vital importance that the thermal conductivity of samples can be kept stable with the rising of the system temperature. As shown in Figure 5, the thermal conductivity at room temperature (RT) is 3.92 W·m^−1^ K^−1^, and the thermal conductivity of the sample decreased with the increasing temperature. The thermal conductivity can still remain at a relatively high level, even up to 383 K. It means that our crystal with a high aspect ratio can be used to achieve a stable, highly efficient laser output.

**Figure 6:**
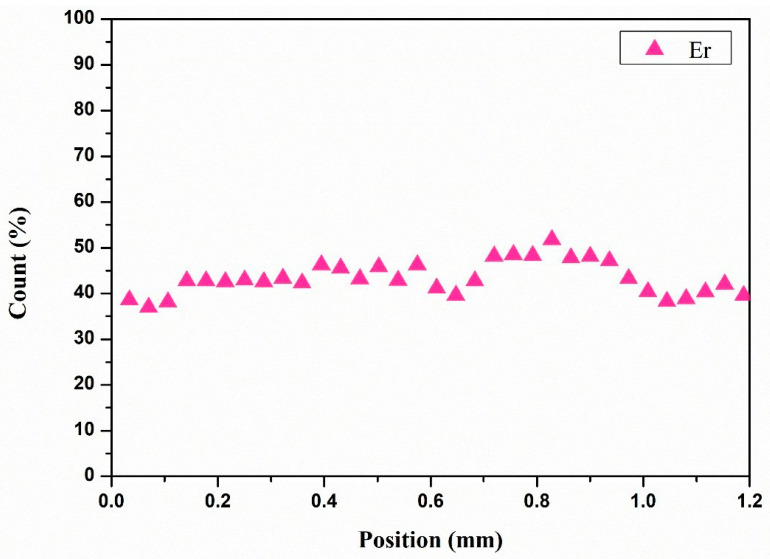
Concentration distribution of Er in 30 at. % Er: YSGG crystal.

The concentration distribution of Er^3+^ can be seen in Figure 6, the distribution of the active ion was homogeneous along the cross section of the sample and where Er^3+^ occupies the position of Y^3+^. Hence, this proved that high doping laser crystals can be grown using the method, and the as-grown crystal can be used to increase the laser output.

**Figure 7:**
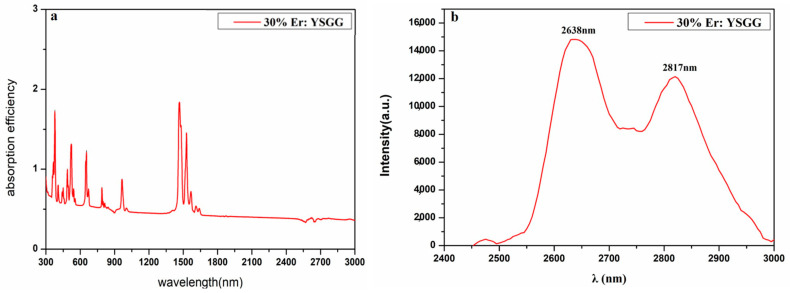
Fluorescence spectra of 30 at% Er: YSGG ((**a**) absorption spectrum; (**b**) emission spectrum).

**Figure 8:**
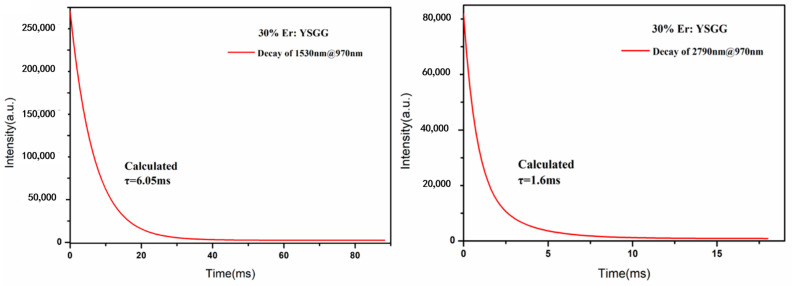
Fluorescence decay curves in 1530 nm and in 2790 nm excited by 970 nm; Er: YSGG: ^4^I_13/2_ (6.05 ms) and ^4^I_11/2_ (1.6 ms).

As we know, the emission band of the high Er-doped crystal is usually centered at the mid-infrared wavelength. We used a spectrophotometer to measure the absorption spectrum of the crystal, and the result is shown in Figure 7a. We found seven absorption peaks at 380, 488, 523, 652, 790, 932 and 1532 nm, and the absorption peak at 932 nm corresponds to the Er3+: ^4^I_15/2_→^4^I_11/2_ transition, which is well matched with commonly used commercial semiconductor pump sources, so the commonly used 970 nm pump source is used for Er^3+^-doped crystals to generate the laser. The fluorescence spectra are shown in Figure 7b; the result of the emission band centered at 2638 nm and 2817 nm clearly reflect the Er: ^4^I_11/2_→^4^I_13/2_ transition in as-grown SCFs. As we know, Er^3+^-doped crystals are used to obtain a laser output around 3 μm and 1.5 μm due to the two main emission peaks located at around 2.79 and 1.5 μm. Compared with a 1.5 μm laser, a 2.79 μm laser is restricted by the difference between the lifetime of ^4^I_13/2_ and that of ^4^I_11/2_. We used a spectrophotometer (FLS980, Edinburgh Instrument, Livingston, UK) to measure the fluorescence decay curve. The dimension of the crystal is Φ 2 × 2 mm^3^, and the end-face of the crystal is carefully polished. According to the mentioned absorption spectrum, the fluorescence decay curve is obtained under a 970 nm LD, the spectral resolution is 0.06 nm, the corresponding filter is added, and the test wavelength is 2400–3000 nm which can reflect the transition. The lifetime of the crystal can be calculated by fitting the curve; the related formula is $\tau_{i}=\frac{\sum \alpha_{i}\tau_{i}^{2}}{\sum \alpha_{i}\tau_{i}}$ ($\alpha_{i}$ is the weight of the decay time; $\tau_{i}$ is the result of the exponential fitting). The lifetime of Er: YSGG has a single exponential fitting. As shown in Table 2, the lower-level lifetime of Er: YAG, Er: GGG and Er: YSGG is 7.25 ms, 4.86 ms and 3.3 ms [18,24]. Therefore, Er: YSGG is a promising material to decrease the self-terminating behavior during the laser operation, so a high-efficiency laser can be obtained. Figure 8 shows the lifetime of the sample a: ^4^I_13/2_ and ^4^I_11/2_ which exerts singly exponential decay. The relevant lifetimes were calculated to be 6.05 ms and 1.6 ms, respectively.

The laser experimental setup (seen Figure 1) was built to explore the laser performance. The pump light was focused on Er: YSGG SCF with a 1:1 coupling system. In order to obtain a high laser output, we used different transmission plane output mirrors (OM) to optimize the system. Moreover, the cooling of the crystal was realized by wrapped it in indium foil, and the crystal was fixed in a copper heat sink. This cooling system can help to remove the heat and keep the temperature at 15 °C.

**Figure 9:**
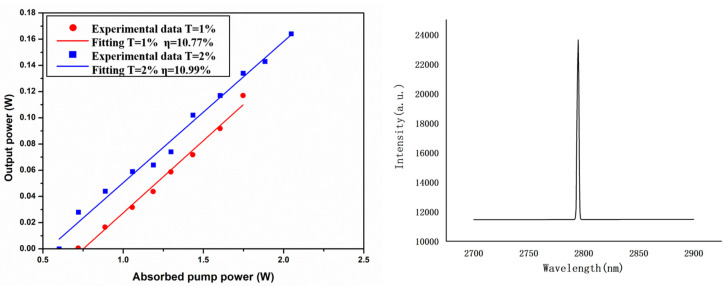
CW laser output at 3 μm.

As can be seen in Figure 9, the maximum output of 166 mW was obtained when the output mirror transmission of 2% was applied, and the central of wavelength was located at 2794 nm. The slope efficiency can be calculated using the ratio of the output power to the absorbed pump power. It is worth mentioning that the absorbed pump power is the efficiently absorbed pump power, so we should subtract the laser threshold. The slope efficiency of the laser output was up to 10.99%, and because of lower hardness and small end face, the SCFs were hard to coat with the antireflection film, which will have influenced the result. On the other hand, as the SCF is air-coated, it will lead to multi-laser modes and a high loss, and the cladding of the crystal fiber can reduce the surface scattering loss and coupling pump wavelength without sacrificing the beam quality. Related research will be needed to verify the superior properties of the single-crystal fiber for achieving a higher power output. Moreover, SCF can be doped with various rare earth elements which can help to improve the output and efficiency in a 3 μm laser of Er-doped host material.

## 4. Conclusions

In this paper, 30 at% Er: YSGG SCFs were successfully grown using the μ-PD method with a <111> oriented YSGG-seed single crystal. The growth parameters were investigated systemically to achieve the high quality SCFs, and the measurement of the as-grown crystal showed great quality and potential applications in laser output. In the following experiment, the max CW laser output was 166 mW and the corresponding slope efficiency was 11%, as demonstrated for Er: YSGG. All of these results indicated that the Er: YSGG SCF can help to optimize the Er-doped laser output, and SCF can be a potential material in future high-power laser systems.

## Figures and Tables

**Table 1:** Composition of Er: YSGG.

	Er: YSGG
Ga	34.6 (wt%)
Y	31.2 (wt%)
Er	22.7 (wt%)

**Table 2:** The fluorescence lifetime of several garnet crystals.

	Er: ^4^I_13/2_ Lifetime (ms)	Er:^4^I_11/2_ Lifetime (ms)	Reference
30 at. % Er: YAG	7.25	0.1	[22]
30 at. % Er: GGG	4.86	0.9	[22]
20 at. %, 5 at. % Er: LuSGG	quenched	0.38	[23,25]
30 at. % Er: SGGM	6.07	0.629	[5]
30 at. % Er: YSGG	6.05	1.6	This work

## Data Availability

Data are contained within the article.
